# Screening of CIMMYT and South Asian Bread Wheat Germplasm Reveals Marker–Trait Associations for Seedling Resistance to Septoria Nodorum Blotch

**DOI:** 10.3390/genes15070890

**Published:** 2024-07-07

**Authors:** Rupsanatan Mandal, Xinyao He, Gyanendra Singh, Muhammad Rezaul Kabir, Arun Kumar Joshi, Pawan Kumar Singh

**Affiliations:** 1 Visiting Scientist, International Maize and Wheat Improvement Center (CIMMYT), Texcoco 56237, Mexico; rup.biotech@gmail.com; 2 Department of Genetics and Plant Breeding, Uttar Banga Krishi Viswavidyalaya, Cooch Behar 736165, India; 3 International Maize and Wheat Improvement Centre, Texcoco 56237, Mexico; x.he@cgiar.org; 4 ICAR-Indian Institute of Wheat and Barley Research, Karnal 132001, India; gyanendra.singh@icar.gov.in; 5 Bangladesh Wheat and Maize Research Institute, Dinajpur 5200, Bangladesh; rezaul.kabir@bwmri.gov.bd; 6 International Maize and Wheat Improvement Center (CIMMYT)-India Office, New Delhi 110012, India; a.k.joshi@cgiar.org; 7 Borlaug Institute for South Asia, New Delhi 110012, India

**Keywords:** Septoria nodorum blotch, *Parastagonospora nodorum*, host resistance, genome-wide association study, marker–trait association

## Abstract

Wheat (*Triticum aestivum* L.) production is adversely impacted by Septoria nodorum blotch (SNB), a fungal disease caused by *Parastagonospora nodorum*. Wheat breeders are constantly up against this biotic challenge as they try to create resistant cultivars. The genome-wide association study (GWAS) has become an efficient tool for identifying molecular markers linked with SNB resistance. This technique is used to acquire an understanding of the genetic basis of resistance and to facilitate marker-assisted selection. In the current study, a total of 174 bread wheat accessions from South Asia and CIMMYT were assessed for SNB reactions at the seedling stage in three greenhouse experiments at CIMMYT, Mexico. The results indicated that 129 genotypes were resistant to SNB, 39 were moderately resistant, and only 6 were moderately susceptible. The Genotyping Illumina Infinium 15K Bead Chip was used, and 11,184 SNP markers were utilized to identify marker–trait associations (MTAs) after filtering. Multiple tests confirmed the existence of significant MTAs on chromosomes 5B, 5A, and 3D, and the ones at *Tsn1* on 5B were the most stable and conferred the highest phenotypic variation. The resistant genotypes identified in this study could be cultivated in South Asian countries as a preventative measure against the spread of SNB. This work also identified molecular markers of SNB resistance that could be used in future wheat breeding projects.

## 1. Introduction

Septoria nodorum blotch (SNB) is a significant foliar fungal disease caused by *P. nodorum* that affects wheat production globally [1]. This fungus prefers the moist and warm areas of the world [2,3], and recently SNB has been reported in India [4,5]. This disease leads to substantial reductions in yield losses and creates an obstacle for wheat breeders striving to develop resistant genotypes [6]. The pathogen mostly infects leaves, resulting in necrotic lesions that reduce their photosynthetic capability and subsequently affect grain yield [7]. The pathogen *P. nodorum* has the ability to generate numerous virulence factors, including *SnToxA*, *SnTox1*, and *SnTox3*, which facilitate its ability to invade wheat plants [8,9]. These factors consist of enzymes that degrade the cell walls of plants, toxins that harm plant cells, and other chemicals that promote fungal growth [1,10]. The genetic heterogeneity of the pathogen enables it to bypass plant resistance mechanisms and adapt to different wheat cultivars [11,12,13,14], while wheat plants possess an intrinsic genetic capability for resistance against SNB [15,16]. Many investigations of SNB resistance, which is often regulated by several genes with minor individual effects, have shown that it is known as quantitative resistance [17,18,19,20,21]. Through the use of advanced genotyping technologies, researchers have conducted an in-depth study of the genetic factors that contribute to SNB resistance. Genome-wide association studies (GWASs) have proved their efficacy in identifying genetic markers associated with different phenotypes, such as SNB resistance, which offers useful insights into the genetic basis of resistance and facilitates marker-assisted selection [22,23,24,25,26,27,28]. Several GWASs conducted on wheat have identified multiple quantitative trait loci (QTL) that are associated with resistance to SNB. These QTLs are dispersed throughout various chromosomes, including 1A, 2A, 3A, 4A, 5A, 6A, 7A, 1B, 3B, 4B, 5B, 6B, 2D, 5D, and 7D [20,21,29,30,31]. GWAS has provided important insights, but issues including genetic variability, population structure, and linkage disequilibrium need to be properly addressed [20,21,22,23,24,25]. The molecular mechanism underlying SNB resistance in wheat may be better understood by combining GWAS data with functional genomics and transcriptomics information. The identification of novel loci and markers linked with SNB resistance genes through GWASs holds great promise for accelerating the development of resistant varieties of wheat, contributing to sustainable wheat production. The aims of the study were to determine the genotypes that are resistant to SNB and to perform a thorough GWAS to find the specific locations that are linked with SNB resistance utilizing 174 wheat accessions from India, Nepal, Bangladesh, and CIMMYT-Mexico.

## 2. Materials and Methods

### 2.1. Plant Materials

One hundred seventy-four spring wheat genotypes were used for the current investigation. Among the accessions, 97 originated from CIMMYT, Mexico, 30 from India, 28 from Nepal, and the remaining 19 from Bangladesh (Supplementary Table S1), which are elite breeding lines and modern varieties in the respective countries.

### 2.2. Disease Screening

Three separate tests were implemented in a greenhouse at CIMMYT-Mexico to evaluate disease severity in wheat seedlings. The experiments were carried out using an RCBD (randomized complete block design), with three replications. The experiments were conducted at temperatures of 22 °C during the day and 18 °C at night, with a 16 h photoperiod. The tests utilized plastic containers as experimental units, with four plants for each entry. The resistant control genotype used was “Erik”, while the susceptible control genotype used was “Glenlea”(Supplementary Table S3). The inoculum was made from the Mexican *P. nodorum* strain MexSn4 (CIMMYT ID: CIMFU-463), which was identified as a ToxA producer based on inoculation tests, filtering experiments, and the ToxA marker assay. Conidiospores were prepared from V8 Juice–PDA medium and were adjusted to 1 × 10^7^ spores mL^−1^ for inoculation, following the protocols described by Hu et al. [32]. Artificial inoculation was carried out when the second leaf had fully grown, at approximately 14 DAP (days after planting). This was carried out by using a hand sprayer to apply the inoculum until it covered the entire plant, with an approximate volume of 0.5 mL per plant. After the leaves had dried, they were moved to a humid chamber with relative humidity of 100% and a temperature of 20 °C to promote infection. After a period of 24 h, the plants were placed back onto the greenhouse bench. The disease scores were evaluated using a linear scale ranging from 1 to 5, where 1 represented the least infected and 5 represented the most infected [32,33,34].

### 2.3. Statistical Analysis

Analysis of variance (ANOVA) was conducted using R software version 4.3.1 with the “metan” package. Additionally, stability and GGE biplot analyses were performed using the “plant breeding” and “GGEBiplotGUI” packages [35,36].

### 2.4. Genotyping

The panel was genotyped at Trait Genetics GmbH, Gatersleben, Germany, using the Illumina Infinium 15 K Bead Chip, which produced a total of 16,028 SNPs. The genotypic data were filtered to exclude SNP markers with minor allele frequencies less than 0.05, unknown chromosome positions, or more than 10% missing SNP data, resulting in 11,184 SNP markers that were included in the subsequent study. Genomic locations of the SNP markers were determined either from the consensus map [37] or via BLAST searches against Chinese Spring reference genome v.1.0 using Phytozome v13 [38].

### 2.5. Population Structure and Principal Coordinate Analysis

The number of subpopulations among the 174 wheat genotypes was estimated using STRUCTURE software v2.3.4, which is a model-based Bayesian cluster analysis tool [39]. The number of presumed groups was set between k = 1 and 10, and the admixture model was employed. Each of the five independent replications of the analysis involved 50,000 burn-in repeats and 10,000 MCMC (Markov Chain Monte Carlo) iterations. The statistical measure ΔK, which is derived from the rate of change in the log-likelihood of the provided data, was employed with Structure Harvester to ascertain the optimal number of clusters [40]. Structure Plot v2.0 was used to create the structure bar plot with the most clusters possible [41]. PCoA was analyzed using the GenAIEx 6.5 software [42].

### 2.6. Kinship and Linkage Disequilibrium Analysis

TASSEL v5.069 was used to analyze all 11,184 SNP markers to generate a kinship matrix as well as clusters among the individual genotypes. A kinship matrix heat map was generated using the R program v4.3.1 [35]. Linkage disequilibrium (LD) of the panel was examined using TASSEL v5.069 [43] and the 11,184 SNP markers. By graphing the R^2^ values against the physical distance (Mb) between the markers using R software, the LD decay distances were obtained for the entire genome and individual chromosomes.

### 2.7. GWAS Analysis for SNB

GLM, MLM, and FarmCPU were employed to identify marker–trait associations. The FarmCPU model was carried out using the R software package rMVP [44], whereas the PCA-based GLM model and the MLM model, which consider both Kinship (K matrix) and population structure (Q value), were executed in Tassel v5.069 [43]. The three experiments were each subjected to a separate GWAS and then examined using the grand mean data across experiments. Significant markers in all models were declared with a threshold of *p* < 0.001.

## 3. Results

### 3.1. Phenotypic Evaluation

**Figure 1:**
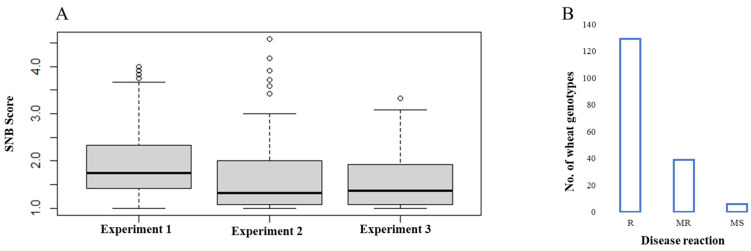
Phenotypic reactions of the tested genotypes. (**A**) Boxplot distribution of SNB scores for the 174 wheat genotypes in individual experiments. (**B**) Histogram distribution of mean disease scores over the three experiments.

The distribution of disease scores in the panel throughout the three experiments was depicted in a box plot diagram (Figure 1A). The examined genotypes showed a skewed distribution towards the resistance side, and around 74% of accessions exhibited resistant reactions (disease scores between 1.0 and 2.0), 39 genotypes showed moderately resistant reactions (2.1–3.0), and only 6 genotypes were moderately susceptible to susceptible, with disease scores higher than 3.1 (Figure 1B, Supplementary Table S1).

Two genotypes (CIM50 and CIM82), which were the most resistant, did not exhibit any infection symptoms in all three experiments, and many other genotypes showed a very low degree of infection symptoms. The 174 wheat genotypes demonstrated a grand mean SNB score of 1.71, and significant correlations were found among individual experiments, ranging from 0.57 (Experiment 1 vs. Experiment 3) to 0.66 (Experiment 2 vs. Experiment 3). The Bartlett test revealed homogeneity of variances among experiments, with *p* < 0.001. ANOVA revealed significant effects in “Genotype” and “Experiment”, as well as their interaction (Table 1). High broad-sense heritability (h^2^_bs_) estimates were obtained for the three experiments, ranging from 91% (Experiment 3) to 95% (Experiment 1).

**Figure 2:**
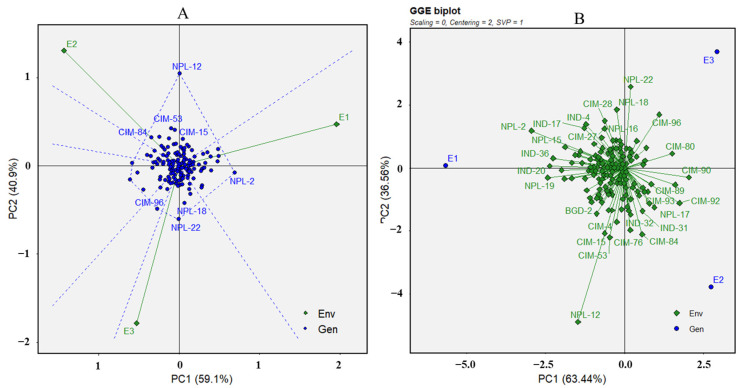
The “which-won-where” view (**A**) and GGE biplot (**B**) based on the G × E interaction of SNB disease scores in three different experiments.

Based on the SNB scores, the three experiments were divided into two sectors with various resistant and susceptible genotypes in the “which-won-where” view of the GGE biplot (Figure 2). The first two principal components accounted for 90.24% of the whole G+GE (Genotype + Genotype × Experiment) variation. 

The three experiments had distinct contributions to the observed difference in SNB resistance, i.e., the first experiment (E1) was more different from the other two experiments (E2 and E3) and made significant contributions to the genotype-by-environment interaction (G × E). Additionally, E1 was situated farther from the origin than E2 and E3, indicating a higher phenotypic variation among genotypes (Figure 2). The tested genotypes displayed varying principal component scores, implying variable genotype-by-environment performance. The lines NPL-12, NPL-17, CIM-22, CIM-51, CIM-83, CIM-87, CIM-97, NPL-8, NPL-28, CIM-39, and NPL-6 demonstrated distinct adaptations for specific environments, as indicated by their performance in experiments E1, E2, and E3. On the contrary, genotypes like CIM-50 and CIM-82 had the lowest principal component values, indicating a reduced genotype-by-environment interaction and increased stability (Figure 2).

### 3.2. Population Structure and Principal Coordinate Analysis

**Figure 3:**
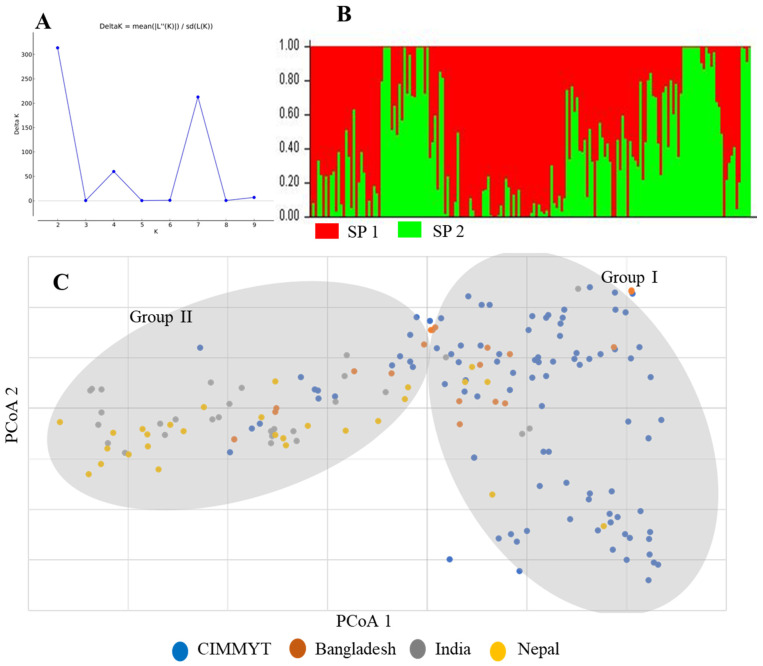
Population structure (**A**,B) and PCoA plot (**C**) of the 174 genotypes.

Based on the ΔK statistic, population structure analysis identified two subpopulations among the 174 genotypes (Figure 3A,B), with 112 and 62 members in subpopulations SP1 and SP2, respectively (Supplementary Table S1). The majority of CIMMYT genotypes (87 out of 97) were found in SP1, whereas most genotypes from Bangladesh (17 out of 19) and those from Nepal (20 out of 28) were found in SP2. Indian accessions did not show obvious differences in the two subpopulations (16 in SP1 and 14 in SP2). Genotypes in SP1 generally exhibited better resistance than those from SP2, according to the mean SNB scores of 1.68 for SP1 and 1.76 for SP2. PCoA analysis also divided the panel into two groups, which were very similar to the two subpopulations identified in the structure analysis (Figure 3C).

### 3.3. Kinship and Linkage Disequilibrium Analysis

**Figure 4:**
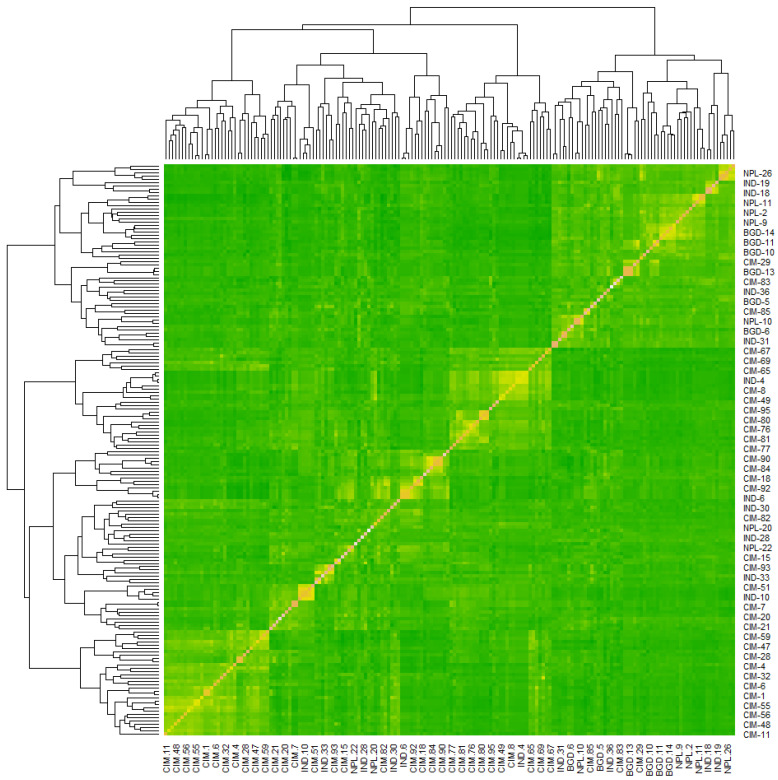
Heatmap and dendrogram of kinship matrix estimated using Van Randen algorithm based on 11,184 SNP markers and 174 wheat genotypes. Yellow colors represent the haploblocks.

Using the 11,184 SNPs, kinship analysis virtually divided the 174 wheat genotypes into two groups, which were very similar to those in the STRUCTURE and PCoA analyses: CIMMYT vs. non-CIMMYT genotypes (as shown in Figure 4). As for the LD analysis, the critical R^2^ value was estimated at 2.24 Mb for the entire genome, and at 2.23 Mb, 4.69 Mb, and 1.78 Mb for the A, B, and D genomes, respectively (Supplementary Figure S1).

### 3.4. Marker Trait Association (MTA) for SNB

**Figure 5:**
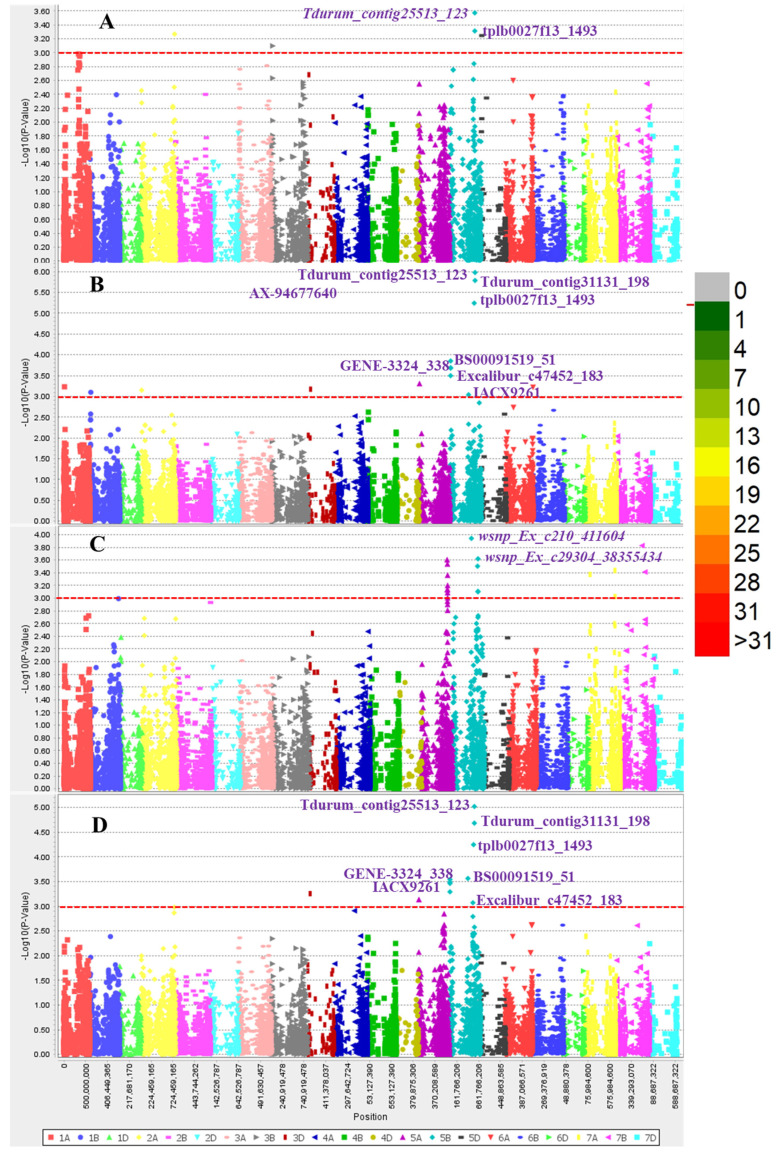
Manhattan plots for MTAs detected from individual experiments such as E1 (**A**), E2 (**B**), E3 (**C**) and the pooled data (**D**). The red horizontal line indicates the significance threshold (*p* = 0.001) used in the present study.

Of the three GWAS algorithms tested in this study, namely GLM, MLM, and FarmCPU, MLM fit the data the best (Supplementary Figure S2) and was thus chosen for subsequent analysis. A total of 11 significant MTAs for SNB resistance were identified (Figure 5), and the SNPs *Tdurum_contig25513_123*, *tplb0027f13_1493*, and *Tdurum_contig31131_198* on chromosome 5B were the most stable and were detected in two individual experiments. The remaining seven MTAs, which were significant in only one of the experiments and the mean data, were distributed on chromosomes 3D, 5A, and 5B (Figure 5). The phenotypic variation explained by these significant markers ranged from 7.35 to 15.66%, with the highest value from *tplb0027f13_1493*, followed by 13.30% from *IACX9261*, and both of these markers are on the long arm of chromosome 5B. There are additional MTAs on this chromosome arm that were significant in this study, and four of them, *tplb0027f13_1493*, *Tdurum_contig25513_123*, *Tdurum_contig31131_198T*, and *IACX9261*, exhibited significant phenotypic effects (Table 2). The genotypes in this panel that exhibited high resistance had a resistance allele for all four markers, as anticipated.

### 3.5. Candidate Genes for the Significant MTAs

Nucleotide sequences of the significant SNP markers were used in BLAST searches against the Chinese Spring reference genome v2.2 available using the Phytozome v13 online tool to identify putative genes associated with disease resistance mechanisms in plants. We employed SNPs for the 11 significant MTAs to explore the putative genes. Each MTA was analyzed using a 2 Mb window to find the putative genes, resulting in a total of eight genes in the genomic intervals (Supplementary Table S2), all of which have known functions relevant to disease resistance.

## 4. Discussion

The potential yield loss caused by SNB is estimated to be up to 16% worldwide, which warrants great efforts regarding resistance breeding and disease management for this disease [45]. Exploring resistant sources in the current wheat panel and employing them in wheat breeding could be a useful approach to managing diseases. In this study, we conducted greenhouse screening for seedling SNB resistance, which avoided the influence of other naturally occurring foliar diseases that mimic SNB symptoms, like spot blotch and tan spot; in addition, greenhouse experiments provide the optimized light and humidity requirements for SNB infection that are often not available for field experiments [32,33,34]. As reported by Thapa et al. [46], greenhouse screening against SNB was effective for the identification of resistant genotypes as well as for the GWAS study.

Using the same panel, Phuke et al. [47] reported high levels of resistance of a CIMMYT germplasm to tan spot, and the present study has drawn a similar conclusion for resistance to SNB (around 74% of genotypes were resistant). However, in a previous study on greenhouse SNB screening of CIMMYT germplasms, only 51 out of 385 lines tested were classified as resistant [48], indicating a significant increase in the frequency of SNB-resistant genotypes in recent CIMMYT germplasms. A possible explanation for this could be the continuous selection for SNB resistance, and the consequent decreased frequency of *Tsn1*, a susceptible gene for multiple wheat diseases, e.g., SNB, tan spot, and spot blotch, which will be further discussed later.

A moderate population structure was found in the current study, which could be ascribed to the fact that CIMMYT wheat germplasms have been widely distributed and utilized globally [47,49], and the frequent and historical germplasm exchange between CIMMYT and the South Asian countries resulted in the reduced population structure observed in this study. LD decay for the A and B subgenomes was smaller than for other spring wheat populations [50,51], and LD for subgenome D was significantly lower than for subgenomes A and B.

In our study, the genomic region on the long arm of chromosome 5B represented by the markers *Tdurum_contig25513_123*, *tplb0027f13_1493*, and *IACX9261* was the most stable, which must represent the *Tsn1* gene that plays a substantial role in SNB resistance [28]. Similar conclusions were made by Al Tameemi et al. [38] using 274 US wheat accessions and by Francki et al. [52] using 232 global wheat accessions. In our study, three MTAs on the short arm of chromosome 5B (*BS00091519_51*, *GENE-3324_338*, and *Excalibur_c47452_183*) were significantly associated with SNB disease, which, based on their physical positions, must represent the *Snn3-B1* locus, which has a significant impact on SNB resistance, according to Downie et al. [26]. It is noteworthy that this genomic region was found to be significant in one of our recent studies on SNB resistance for a panel of 296 Indian genotypes [53]. The significant MTAs identified on chromosomes 3D (*AX-94978939*) and 5B (*Tdurum_contig31131_198*) do not match with previously identified QTL; therefore, these may be novel genomic regions for SNB resistance, but further validation is needed before they are utilized in breeding.

Resistance breeding in wheat is a tedious task, mainly due to the quantitative nature of resistance, which is true for leaf spotting diseases including SNB, as well as for adult plant resistance against rusts and powdery mildew [54]. This, together with other important quantitative traits like yield potential and abiotic stress resistance, often slows progress regarding the genetic gain of wheat breeding. Nevertheless, the existence of multi-disease resistance genes/loci provides breeders an opportunity to incorporate a single gene/locus for multiple traits, like *Lr34*/*Yr18*/*Sr57*/*Pm38*/*Sb1* and *Lr46*/*Yr29*/*Sr58*/*Pm39*, which should be fully utilized by breeders. *Tsn1* is one such gene, which was initially associated with resistance against SNB and tan spot, and later spot blotch, too [53]. For the time being, the former two diseases are not important in South Asia, but spot blotch has been a major disease in the region and accounts for significant annual yield losses [55]. Considering this, it is strongly recommended that wheat breeders in South Asia eliminate this susceptible gene from their breeding materials to first increase spot blotch resistance and then be prepared for possible future epidemics of SNB and tan spot. If an SNB epidemic occurs in the region in the future, which is not unlikely considering the changing climate and the wide adoption of conservation agricultural practices in the region, additional SNB resistance genes/loci could be incorporated, among which are the resistance alleles for the markers identified in the current study, especially *Snn3-B1* on chromosome 5B, which has shown importance in multiple studies.

## 5. Conclusions

In conclusion, a panel of 174 wheat genotypes from CIMMYT and South Asian countries was evaluated in this study for seedling resistance against SNB. About 74% of genotypes exhibited resistant reactions, which could be ascribed to the low frequency of the susceptibility gene *Tsn1.* Using the MLM algorithm for GWAS, 11 MTAs were identified, representing the known susceptibility genes *Tsn1* and *Snn3-B1*, as well as two potentially novel MTAs on chromosomes 3D and 5B that confer SNB resistance. The identified MTAs could be useful for marker-assisted selection in wheat breeding using the resistant genotypes identified in this study as donors.

## Figures and Tables

**Table 1:** Analysis of variance (ANOVA) for SNB resistance in the GWAS panel.

Source	Df	MS	*p*-Value
Experiment	2	18.38816	*p* < 0.001
Replication	6	0.128591	*p* > 0.05
Genotype	173	3.042647	*p* < 0.001
Genotype × Experiment	346	0.553166	*p* < 0.001
Residual	1038	0.092841	

**Table 2:** Markers significantly associated with seedling SNB resistance using the MLM algorithm.

Exp.	Marker	Chr	Position	*p*-Value	R^2^ (%)
Exp2	*AX-94677640*	5B	6,838,168	2 × 10^−4^	8.96
Mean	*AX-94677640*	5B	6,838,168	3 × 10^−4^	8.48
Exp2	*AX-94864577*	5B	6,974,754	1 × 10^−4^	9.68
Mean	*AX-94864577*	5B	6,974,754	3 × 10^−4^	8.63
Mean	*AX-94978939*	3D	52,806,848	6 × 10^−4^	7.65
Exp2	*AX-94978939*	3D	52,806,848	7 × 10^−4^	7.35
Exp2	*BS00091519_51*	5B	6,648,517	2 × 10^−4^	8.80
Mean	*BS00091519_51*	5B	6,648,517	3 × 10^−4^	8.44
Exp2	*Excalibur_c47452_183*	5B	6,654,116	3 × 10^−4^	8.22
Mean	*Excalibur_c47452_183*	5B	6,654,116	5 × 10^−4^	7.83
Exp2	*GENE-3324_338*	5B	3,521,225	5 × 10^−4^	7.76
Mean	*GENE-3324_338*	5B	3,521,225	5 × 10^−4^	7.40
Exp2	*IACX9261*	5B	546,703,936	6 × 10^−4^	13.30
Mean	*IACX9261*	5B	546,703,936	6 × 10^−4^	10.31
Exp2	*Tdurum_contig25513_123*	5B	565,753,503	2 × 10^−6^	15.23
Mean	*Tdurum_contig25513_123*	5B	565,753,503	1 × 10^−5^	12.93
Exp1	*Tdurum_contig25513_123*	5B	565,753,503	3 × 10^−4^	8.59
Exp3	*Tdurum_contig31131_198*	5B	417,443,545	1 × 10^−4^	11.34
Mean	*Tdurum_contig31131_198*	5B	417,443,545	3 × 10^−4^	9.49
Exp2	*Tdurum_contig31131_198*	5B	417,443,545	9 × 10^−4^	7.86
Exp2	*tplb0027f13_1493*	5B	565,754,215	1 × 10^−6^	15.67
Mean	*tplb0027f13_1493*	5B	565,754,215	2 × 10^−5^	11.77
Exp1	*tplb0027f13_1493*	5B	565,754,215	2 × 10^−4^	7.68
Exp3	*wsnp_Ex_c210_411604*	5A	584,054,461	8 × 10^−4^	7.50
Exp3	*wsnp_Ex_c29304_38355434*	5A	584,451,511	1 × 10^−3^	7.58

## Data Availability

The original contributions presented in the study are included in the article/Supplementary Materials, further inquiries can be directed to the corresponding author.

## Supplementary Materials

The following supporting information can be downloaded at https://www.mdpi.com/article/10.3390/genes15070890/s1. Supplementary Table S1: List of genotypes with their phenotypic values in three experiments, and Q values from the structure analysis; Supplementary Table S2: Transcript id and functional annotation of MTAs; Supplementary Table S3: SNB disease scores of check genotypes for three experiments and their pooled; Supplementary Figure S1: LD decay plots for whole genomes and individual chromosome; Supplementary Figure S2: QQ plots for GWAS using GLM, MLM and FarmCPU models.

## Abbreviations

ANOVA	Analysis of Variance
FarmCPU	Fixed and random model Circulating Probability Unification
GWAS	Genome-Wide Association Study
GLM	General Linear Model
MLM	Multiple Linear Model
MTA	Marker Traits Association
QTL	Quantitative Trait Loci
rMVP	Memory-Efficient, Visualize-Enhanced, Parallel-Accelerated GWAS Tool
SNB	Septoria Nodorum Blotch
SNP	Single Nucleotide Polymorphism
