# Supplementary figures and images for: Screening of CIMMYT and South Asian Bread Wheat Germplasm Reveals Marker–Trait Associations for Seedling Resistance to Septoria Nodorum Blotch
Source: Genes (Basel). 2024 Jul 7;15(7):890. doi: 10.3390/genes15070890 (PMC11276481; doi:10.3390/genes15070890)

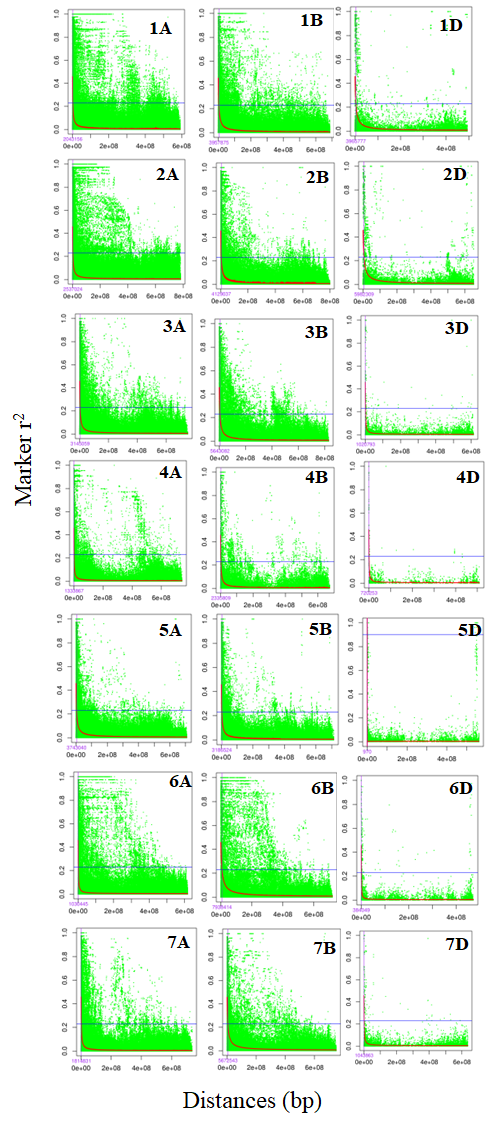

Supplement: Supplementary file 1 [file genes-15-00890-s001.zip › Supplementary Figure 1.png]

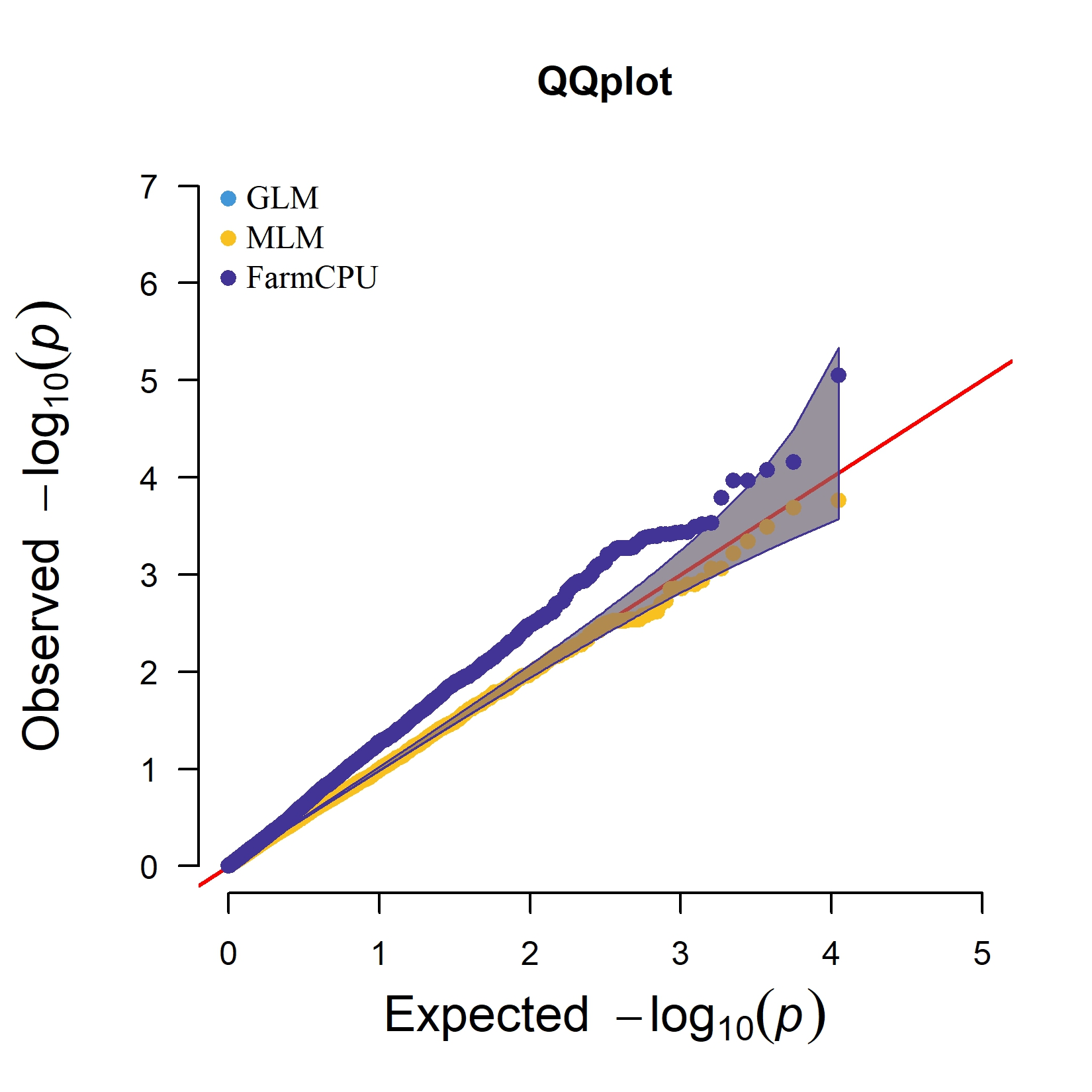

Supplement: Supplementary file 1 [file genes-15-00890-s001.zip › Supplementary Figure2.png]
